# Fluoxetine plus lithium for treatment of mental health impairment in Long Covid

**DOI:** 10.1007/s44192-022-00027-w

**Published:** 2023-01-03

**Authors:** Jeffrey Fessel

**Affiliations:** Department of Medicine, University of California, 2069 Filbert Street, San Francisco, CA 94123 USA

**Keywords:** SARS-CoV-2, Long Covid, Mental symptoms, Treatment, Fluoxetine lithium

## Abstract

**Purposes:**

(1) To summarize the mental conditions that may accompany persistent symptoms following acute infection by SARS-CoV-2, often termed Long Covid; (2) to formulate treatment based upon the brain cells that are dominantly affected.

**Methods:**

(1) Review the reports relating to the mental symptoms occurring in Long Covid. (2) Review the drugs that address the brain cells affected in Long Covid, and suggest pharmacotherapy for those patients whose response to psychotherapy is suboptimal.

**Results:**

Long Covid affects ~ 10% of patients infected by SARS-CoV-2, and mental symptoms affect ~ 20% of persons with Long Covid. The brain cell-types that have been demonstrated as dominantly affected in Long Covid are astrocytes, oligodendrocytes, neurons, endothelial cells/pericytes, and microglia. Lithium and fluoxetine each address all of those four cell-types. Low dosage of each is likely to be well-tolerated and to cause neither clinically important adverse events (AE) nor serious adverse events (SAE).

**Conclusion:**

For those patients whose response to psychotherapy is suboptimal, lithium and fluoxetine should be administered in combination for both depth of benefit and reduction of dosages.

## Introduction

### Global extent of mental symptoms in persons with Long Covid

Starting 12 weeks after their initial infection with SARS-CoV-2, many patients have symptoms that they did not have prior to the infection, and that then persist for weeks or months, a condition often termed ‘Long Covid’. Data collected in 2022, by the U.S. Census Bureau, showed that 1 in 13 adults in the U.S. (7.5%) have long COVID symptoms. Similarly, the UK Office of National Statistics reported 23.6 million cases of SARS-CoV-2 infection in the UK, and 1.2 million of those (5.1%) said that they had symptoms lasting for more than 12 weeks. Chen et al synthesized information from 50 studies involving almost 1.7 million people, showing that 43% still had symptoms 28 days after the acute infection with Covid-19 [1].

### Mental symptoms in persons with Long Covid

In this article, the term ‘mental symptoms’ refers to the following: ‘brain fog’, implying difficulties with concentration or memory; anxiety; cognitive impairment as assessed either subjectively or objectively; dementia; depression; psychotic symptoms; and symptoms of post-traumatic stress disorder (PTSD). Note that the following reports are of symptoms present for ≥12 weeks after the acute SARS-CoV-2 infection.

#### Concentration or memory impairment

One large survey showed 17.9% of 886 persons with memory impairment, and 25.9% of 254 persons with concentration impairment [2]. In another large survey, involving 57 studies with 250,351 survivors of Covid-19 infection, Groff et al. found that 54% had symptoms ≥ 6 months after the acute infection, and 23.8% had difficulty in concentration [3]. The ComPaRe Long Covid prospective cohort showed that ‘brain fog’/difficulty concentrating, had a prevalence at 60 days of 71.8%, and at 360 days of 60.2% [4]. A report involving 10,530 subjects, showed brain fog in 32% and memory issues in 28% [5]. Studies of smaller numbers of patients showed 39.8% of 236 persons with memory complaints [6], and brain fog affecting 37% of 113 persons [7]. At 12 months after their acute infection, only 22.9% of 96 subjects were symptomless, 39.6 had difficulty concentrating, and 32.3% had word-finding difficulty [8]. A report from South Korea is noteworthy because it specified that > 99% of subjects had no underlying psychiatric disease, and found that concentration difficulties affected ~ 32% at 3 months and persisted at 1 year in ~ 23% [9] Also at 1 year’s follow-up of 120 persons, memory loss affected 34% and problems concentrating affected 28% [10].

#### Anxiety

Among the subjects mentioned in the above studies, anxiety affected 36.9% [6] (Tize…), 35.6% [11], 34% [12], ~ 30% [3], 23% [5], and 18.7% [2]; and anxiety with depression 33% [7]. For 900 subjects followed for six months, ~ 30% had anxiety, which fell to ~ 17% for the 241 followed for 2 months [9]. The 1276 subjects who completed six and twelve month follows-up, had anxiety/depression at 6 months that was slight, moderate, or severe, in 20%, 3%, and 1%, and at twelve months was 22%, 4%, and 0 [13].

#### Cognitive impairment as assessed either subjectively or objectively

A systematic review of 57 studies involving 50,351 participants, showed 18% with cognitive impairment [3]. Another systematic review, this of 43 studies involving 13,232 subjects, showed cognitive impairments in 22.2% after ≥ 12 weeks of follow-up [14]*.* A meta-analysis included six reports, showing that 24.3% (range 9–66%) of 727 subjects had objectively measured, cognitive dysfunction [12]. 6 months after acute infection with SARS-Cov-2, ~ 29% of 900 participants had cognitive dysfunction, which fell to ~ 22% in the 241 who had a twelve month follow-up [9]. 395 individuals who survived acute infection had a strictly defined, new neurological diagnosis (group 1), and were matched with 395 survivors of acute infection but who had no new neurological diagnosis (group 2); both groups completed a modified Rankin scale (mRS), which assesses disability and is compared over time to check for recovery and degree of continued disability [15] (a score of 0 is no disability, 5 is disability requiring constant care for all needs, 6 is death). For both groups, baseline mRS score was 0 but at six months scores for group 1/group 2 subjects were the worst: score 0, 15%/22%, score 4, 28%/20%; score 5, 8%/4%; OR was 1.98 (P < 0.02) in a multivariable regression analysis that included histories of dementia (group 1, 14%; group 2, 6%) or psychiatric illness (group 1, 16%; group 2, 9%).

#### Dementia

It is no surprise that, despite substantial rates of cognitive impairment, frank dementia as a consequence of SARS-CoV-2 infection is seldom reported, because even if its degree qualifies as Mild Cognitive Impairment, (MCI) the progression of MCI to Alzheimer’s dementia is slow: McGirr et al. reported that after 3 years, which is the duration of the SARS-CoV-2 pandemic, only 33.6% of subjects with MCI progressed to Alzheimer’s dementia (AD), and their mean age was 75 years [16]. Further, the prevalence of AD at age 75 is about 10%, so it would require a very large cohort of survivors of SARS-CoV-2 infection who are that old, to show that dementia is more than expected. Using their electronic health records, Taquet et al. analyzed outcomes at six months, of 236,379 survivors of SARS-CoV-2 infection followed for 6 months; their mean age was 46, those with a history of dementia were excluded, and 0.67% developed a new diagnosis of dementia [17]. Their published data do not show the dementia rate by decade of age but the mean age of those with encephalopathy was 66.7 and their HR for dementia was 4.25 versus those without encephalopathy; the rate of dementia in those with encephalopathy was 5.75-fold higher than that of a matched cohort without encephalopathy. A limitation of the report by Taquet et al. is that the diagnoses could not be verified.

It is also notable that there is an increased rate of SARS-CoV-2 infection of astrocytes and neurons that are homozygous for APOE4 as compared with those cell-types that are homozygous for APOE3 [18]. Further, SARS-CoV-2 infection caused higher mortality in mice bearing the APOE4 variant than in mice bearing the APOE3 variant, which was also the case for patients infected with SARS-CoV-2 in the UK Biobank [19]. Also indicating a propensity to AD, single cell transcriptome analysis showed that in AD subjects their brain endothelial cells had elevated levels of *BSG* and *Furin* relative to neurons and other brain cell-type, presumably accounting for the known cerebral microvascular disease in AD [20]. A review by Maiese et al. showed results in 197 brains, with microthrombi in 10.6%, micro- and perivascular hemorrhages in 12.2%, and ischemic lesions in 15.2% [21].

#### Depression

In the report by Taquet et al., a first diagnosis of mood disorder affected 4, 22% but was approximately doubled in those older subjects who had encephalopathy [17]. In many other reports, the prevalence of depression showed a wide variation in prevalence from 8% at three months [2] to 46.9% at six months [11]; the mean prevalence in the 11 reports was 29.0%, median 26.5% [2, 3, 5–7, 9, 11, 13, 15, 17, 22].

#### Schizophrenia-like symptoms

The only report of a first diagnosis of psychotic disorder is that by Taquet et al., affecting 0.42% but approximately tripled in those older subjects who had encephalopathy [17].

#### Symptoms of post-traumatic stress disorder

There are five reports of PTSD in persons with SARS-CoV-2 infection, affecting 9.14% [2], ~ 15% [3], 35% [7], 30% [23], 37.2% [11].

### Pharmacotherapy for the mental problems associated with Long Covid

Despite the above substantial prevalence’s, and the need to help many patients whose response to psychotherapy is suboptimal, there is not yet a recommended, effective pharmacotherapy for these mental problems. That is primarily because many genetic, metabolic, and environmental factors have been associated with the condition but pharmacotherapy for all of them would entail using an impractical number of drugs. Nevertheless, considering the extent of the problem, an effective drug treatment would be advantageous. A simplified and rational approach to formulating a drug regimen for control of the mental problems associated with Long Covid, would be to ascertain which of the brain cell-types are dominantly affected and to direct therapy toward them because, as shown here, there are available drugs that address some or all of the five main brain cell-types. Evidence provided below, shows that the cognitive impairment in Long Covid is due to reduced numbers of astrocytes, myelinating oligodendrocytes, neurons, and endothelial cells, plus increased numbers of microglia. Treatment with lithium plus fluoxetine, also described below, benefits each of those abnormalities, and should alleviate the mental disturbance of Long Covid. Clinical trial would demonstrate the validity or otherwise, of this proposal.

## Infection of astrocytes, oligodendrocytes, neurons, and endothelial cells/pericytes by SARS-CoV-2

### Receptors for SARS-CoV-2 allowing infection of cells in brain and elsewhere

The major portal of entry into cells by SARS-CoV-2 is mostly via the angiotensin-converting enzyme 2 (ACE2) receptor; however, that receptor has a variable presence in the brain. As will be shown, both Crunfli et al. and Andrews et al. demonstrated infection of astrocytes but neither of them could demonstrate that astrocytes have ACE2 receptors [24, 25]. Crunfli et al. found that neuropilin receptor 1 (NRP1) allows entry for SARS-CoV-2 [7]; and Andrews et al. found that astrocytes could be infected by the SARS-CoV-2 virus via receptors for CD147 and DPP4 [25]. Despite the above reports showing no ACE2 receptors in astrocytes, Song et al. found that neuronal infection of human brain organoids could be prevented by blocking ACE2 with antibodies, demonstrating that, in fact, ACE2 does occur on neurons, if only sparsely [26]. Chen et al. confirmed this by analyzing data from publicly available brain transcriptome databases, showing no ACE2-expressing nuclei in the prefrontal cortex but it was expressed in other brain locations as well as in excitatory and inhibitory neurons, astrocytes, oligodendrocytes, and endothelial cells [27]. The apparently conflicting data may be either because the brain contains several genetic variants of ACE2, that determine a differential response to SARS-Cov-2 infection [28], or because ACE2 receptors are present in some brain cell-types and not others.

### Astrocytes

Crunfli et al. used three different antibodies against the spike S1 component of the SARS-CoV-2 virus, in order to demonstrate the presence of that virus [24]. In one of the five brains examined by Crunfli et al., approximately 70% of astrocytes contained the virus. Crunfli et al. also analyzed the proteome of both infected astrocytes and Covid-19 postmortem brain tissue, showing that glycolysis/gluconeogenesis, carbon metabolism, and the pentose phosphate pathways, were the most involved. Collectively, their data demonstrate that in the brain, SARS-CoV-2 affects energy metabolism and modulates proteins associated with neurodegeneration.

### Myelinating oligodendrocytes

During myelination by oligodendrocytes an extension of its plasma membrane, containing myelin oligodendrocyte glycoprotein (MOG) and myelin basic protein (MBP), wraps itself around a naked axon and ensheathes it with multiple layers of myelin proteins. Undifferentiated oligodendrocyte precursor cells (OPC) have markers for PGDF and Olig2; both OPC and mature, myelinating oligodendrocytes express chemokine receptors CXCR1, CXCR2, and CXCR3. Mature myelinating oligodendrocytes also express MOG and MBP. Reduced numbers of myelinating oligodendrocytes, causing impaired neuronal myelination, cause abnormal neural tracts and consequent cognitive impairment.

Several reports show that in SARS-CoV-2 infection, one reason for low numbers of myelination oligodendrocytes, is the formation of antibodies directed against them. Young et al. saw antibodies against PDGF correlating with the severity of SARS-CoV-2 infection [29]; Schwabenland et al. found antibodies against Olig-2 [30]; Manzano et al. saw anti-MOG seropositivity in 6.7% of patients [31]; and Wang et al. documented plasma antibodies against CXCR1, and CXCR3 in 194 infected persons [32]. Ide et al. reported one case and reviewed five other reports of single cases; all of them had anti-MOG antibodies with symptoms related to involvement of brain, spinal cord, or optic nerve [33].

### Neurons

Although anosmia is a frequent symptom in patients with infection by SARS-CoV-2, and sometimes in otherwise asymptomatic persons, its cause is uncertain. It could arise either from involvement of nasal epithelium, or from neuronal infection. Khan et al. studied 85 cases who had died a few days after infection, and postulated that transient, insufficient support from sustentacular cells in the nasal mucosa, created transient olfactory dysfunction; they did not find evidence for infection of olfactory sensory neurons [34]. Matschke et al. examined 40 brains and found SARS-CoV-2 viral proteins in cranial nerves originating from the lower brainstem, none in the olfactory nerve, and only in isolated cells of the brainstem; interestingly, the presence of SARS-CoV-2 in the brain was not associated with the severity of neuropathological changes [35]. In fact, only sparse reports indicate infection of brain neurons: in one brain examined by Crunfli et al. approximately 20% of neurons contained the virus [5]; and Paniz-Mondolfi et al. saw viral particles in neurons of one case [36]. One possible reason why, despite the frequency of neuropsychiatric symptoms, direct neuronal infection is minimal, might be because neurons are indirectly affected. Thus, Charnley et al. noted that the nucleocapsid protein of the virus contains a number of highly amyloidogenic short peptide sequences, and that open reading frame (ORF) proteins are good candidates for amyloid formation in vivo; in fact ORF6 is the most cytotoxic single protein of the SARS-CoV-2 proteome [37]. By using bioinformatic screening, they identified two peptides from the SARS-CoV-2 proteome, one from ORF6 and one from ORF10, and those peptide sequences self-assembled into amyloid, causing toxicity to neuronal cells. Charnley et al. suggested that, as an indirect effect of SARS-CoV-2 infection, cytotoxic aggregates of SARS-CoV-2 proteins may trigger neurological symptoms. Other indirect data come from the studies described below, showing impairments in myelinating oligodendrocytes whose consequences affect neuronal myelination and, thus, neuronal function and cognition [38].

### Endothelial cells/pericytes

The data reported by Crunfli et al. also suggest that SARS-CoV-2, by occupying and perhaps activating neuropilin receptors (NRPR) in the brain, might adversely affect the cerebral microcirculation because NRPRs also bind the proangiogenic factors VEGF, PDGF, and HGF/SF [24]. In fact, in four of the five brains from persons who had died from SARS-CoV-2 infection examined by Crunfli et al., four had microvascular damage, produced by inflammatory cells invading endothelium in two, by capillary damage in one, and by perivascular edema in one. Kirschenbaum et al. examined the brains from six persons who had died from SARS-CoV-2 infection; all had thrombus in the cerebral microcirculation which, in one subject showed intra-endothelial lymphocytic infiltration [39]. Taha and Samavati analyzed 21 studies with a total of 1159 patients and found that among patients hospitalized for SARS-CoV-2 infection, 46.8% had one or more antiphospholipid antibodies [40]. Del Papa et al. noted a close association between antiphospholipid antibodies and anti-endothelial antibodies [41]; that was confirmed by Shi et al., who found that IgG (presumably containing antibodies) derived from sera of patients hospitalized with COVID-19, induced activation of cultured endothelial cells [42]. These results suggest strongly that antibodies promoted by SARS-CoV-2 infection, affect endothelial cell function. Pericytes are also involved: Bocci et al. demonstrated that pericytes have the ACE2 receptor and that cells of cerebral capillaries from a SARS-CoV-2 infected patient showed viral RNA, which is a possible source of entry into the brain by SARS-CoV-2 [43]. Hirunpattarasilp et al. induced capillary constriction by exposing human cortical slices to SARS-CoV-2 spike protein, resulting in decreased conversion of vasoconstricting angiotensin II to vasodilating angiotensin I; that was shown as caused by the occupation of ACE2 by SARS-CoV-2 because the effect from blocking angiotensin1 mimicked the effect from blocking ACE2 [44].

### Microglia

Thakur et al. studied 28 brains, assessing the presence of SARS-CoV-2 by RT-qPCR, RNAscope, and immunocytochemistry using primers, probes and antibodies directed against the spike and nucleocapsid regions [45]. Low to very low yet detectable, viral RNA levels were in the majority of brains; and each had microglial activation, and microglial nodules, most prominently in the brainstem. However, Andrews et al. saw only a minimal increase of microglia during infection by SARS-CoV-2 [25].

## In sum, in Long Covid, reduction in numbers of astrocytes, oligodendrocytes, neurons, and endothelial cells, and increase in numbers and activity of microglia, may occur because of infection by SARS-CoV-2. Shown below, treatment with lithium plus fluoxetine addresses all of these abnormalities

### Abnormalities of brain cell-types addressed by lithium

#### Astrocytes

Using cultures of optic nerves, Rivera and Butt showed that lithium caused a doubling of astrocyte numbers (P < 0.001) [46].

#### Oligodendrocytes

Meffre et al. found that lithium stimulated maturation of oligodendrocytes, which promoted remyelination after lysolecithin-induced demyelination of organotypic cerebellar slice cultures [47].

#### Neurons

Lithium promoted neurogenesis by inducing increased levels of both BDNF and GDNF in neuronal and astrocyte cultures [48]; and increased neurogenesis (p < 0.001) was seen in the dentate gyrus of rats treated for 28 days with lithium by Son et al. [49]. Likewise, there was an approximately 25% rise in cells staining with BrdU, which stains proliferating cells, in the dentate gyrus of rats given treatment with lithium in an amount that gave a blood level in the therapeutic rage for humans [50]. AD-model mice have decreased hippocampal neurogenesis, that was reversed by lithium [51]. Studies by Dwivedi et al. showed that addition of lithium to cultures of hippocampal neurons halved neuronal death caused by glutamate; dendritic length increased by 44%; expressions of anti-apoptosis factors Bcl-2 and Bcl-xL were increased (p < 0.001); and pro-apoptosis factors BAD, Bax, and caspase 3, were decreased (p < 0.001 [52].

Lithium benefits mitochondria; it increased the number and size of neural mitochondria, and their cytochrome c content [53]; it reduced oxidative stress, as reflected by lower levels of ROS, 4-HNE, and protein carbonyls; it increased levels of the antioxidants catalase, heme-oxygenase and glutathione [54]; and it raised the content of superoxide dismutase (SOD) in neuronal cell cultures [53]. Excessive Ca^2+^ and opening of the MTP are adverse for mitochondria, leading to the release of cytochrome c and neuronal death; those effects were minimized by lithium, as shown by Maurer et al. who incubated homogenates of brain from heathy individuals with lithium at a concentration of 1 mM/L and also saw increased activity, as compared with controls, of complexes I and III by 165% (p = 0.03) and of complexes II and III by 146% (p = 0.00002) [55]. Likewise, Shalbuyeva presented data showing that lithium prevented the MTP from opening and allowing entry of excess Ca^2+^ [64]. Finally, lithium stimulates mitophagy, that both removes damaged mitochondria and promotes mitochondrial biogenesis [56].

#### Endothelial cells

Ji et al. demonstrated that lithium increased the integrity of the blood brain barrier by 46% (P = 0.006), one mechanism for which was to increase the protein levels of the tight junctions between adjacent endothelial cells, mediated by Claudin 5 and ZO-1 [57]. Lithium also induced proliferation and migration of cultured endothelial cells [58].

#### Microglia

The microglial activation caused by adding LPS to microglial cultures, was inhibited by lithium which, therefore, has anti-inflammatory effect in the brain [59]. Yuskaitis and Jope showed that lithium diminished the microglial migration induced by GSK3 [60]. P53 is a nuclear transcription factor that is critical for activating the expression of genes involved in cell-cycle arrest and stress-induced apoptosis. Davenport et al. showed that lithium reduced p53 expression by microglia and prevented microglia-induced neurotoxicity; in addition, the release by microglia of TNFα that was induced by lipopolysaccharide (LPS), was also significantly reduced by lithium [61]. Activated microglia express C′3 receptors and lithium treatment of microglia induces them to secrete C′39 [62], which might be a neuroprotective mechanism [63]. The microglial activation caused by adding LPS to microglial cultures, was inhibited by lithium which, therefore, has anti-inflammatory effect in the brain [59]*.* In brief, lithium inhibits microglial activation via several mechanisms and, therefore, has anti-inflammatory activity in the brain.

### Abnormalities of brain cell-types addressed by fluoxetine

#### Astrocytes

Kinoshita et al. demonstrated that fluoxetine increased the release of ATP by astrocytes that, in turn, increases astrocytic production of BDNF [64].

#### Oligodendrocytes

Fluoxetine prevented the apoptosis of oligodendrocytes that is mediated by expression of pro-nerve growth factor and its neurotrophin receptor [65]; and it also prevented the reduction of oligodendrocytes caused in rats by chronic unpredictable stress [66].

#### Neurons

The inhibitory effect of stress upon neurogenesis in the dentate gyrus was counteracted by fluoxetine [67]. Benefit to cognition from improved neuronal numbers or function, largely depends upon its effect on neural tracts. Bianchi et al. used high-performance liquid chromatography and fluorescence detection to quantify alpha-tubulin isoforms and showed that the neuron-specific delta2-tubulin was increased by chronic fluoxetine [68]; it is likely that the result of such cytoskeletal changes would be reflected by improvement in neural tracts which then might benefit cognition. Stanisavljevic et al. used immunohistochemical detection of c-Fos protein expression, to detect activated neuronal circuits in rats subjected to chronic isolation stress; fluoxetine increased activation in the striatum significantly more than in control [69].

Despite the above positive studies, there are occasional ones that did not show uniform, neuronal benefit from fluoxetine. For example, Ma et al. found that fluoxetine alleviated the impaired spatial learning of middle-aged APP/PS1 mice [70] but, perhaps surprisingly, the same investigative group saw no benefit from fluoxetine on the decreased volume and length of myelinated fibers of mice that had been subject to chronic stress [71]

#### Endothelial cells

After cerebral arteriolar thrombosis had been induced, those arterioles as well as the adjacent ones without thrombosis, became dilatated when infused with fluoxetine; this effect was shown to be due to inhibition of the hydrolysis of acetylcholinesterase, thus enhancing cholinergic activity [72]. Interestingly, the induced vasodilatation by fluoxetine was independent of serotonin, as shown by infusion of fluoxetine together with methylsergide, which blocks the serotonin receptor.

#### Microglia

Fluoxetine prevented microglial activation [65], and like lithium, it does so via several mechanisms. Chung et al. found that in microglial cultures, fluoxetine inhibited expression of proinflammatory cytokines and of inducible nitric oxide synthase; and it attenuated microglial NADPH oxidase activation, production of reactive oxygen species and reactive nitrogen species with consequent oxidative damage [73]. Likewise, Zhang et al. saw significant inhibition by fluoxetine of LPS-induced activation of microglia and the subsequent release of multiple pro-inflammatory and cytotoxic factors including tumor necrosis factor-α, interleukin-1β, nitric oxide, and reactive oxygen species [74]. Lee et al. also saw significant inhibition by fluoxetine of the microglial activation that had been induced by spinal cord injury [65]. Concordant with decreased expression of proinflammatory cytokines, fluoxetine down-regulated M1 activation and up-regulated M2 activation in both microglial primary cells and in derivative cell lines [75]. It also decreased the release of glutamate and d-serine from LPS-activated microglia, producing an increase in the survival of co-cultured cortical neurons that had been deprived of glucose and oxygen. In brief, fluoxetine inhibits microglial activation and, therefore, has anti-inflammatory activity in the brain.

### Adverse effects

The choice of a medication is partly determined by its safety profile, particularly for serious adverse events (SAEs). Following are summaries of reports of SAEs for the two drugs.

#### Lithium

SAEs, include hypercalcemia, hypothyroidism [76], nephrogenic diabetes insipidus, and renal insufficiency [77]. Low dosage (< 100 mgs daily) benefited Alzheimer’s dementia; thus 75 mg daily might be an appropriate dose in Long Covid. It should not be used if glomerular filtration rate is < 60 mL/min.

#### Fluoxetine

Beasely et al. obtained data from 25 double-blind clinical trials involving 4016 patients with MDD randomized to treatment that included fluoxetine 20 to 80 mg/d [78]. At a dose of 20 mg/d, fluoxetine-treated patients had a discontinuation rate due to adverse events that was not statistically significantly different from that in placebo recipients. In this article, the suggested dose is 10 mg/d in combination with lithium.

## Discussion

This article suggests that those patients with Long Covid who have mental symptoms but a suboptimal response to psychotherapy, may benefit from the addition of rational pharmacotherapy. Such non-response to psychotherapy is not rare. In a recent report, Woodbridge et al. analyzed 28 studies involving 2436 participants with Borderline Personality Disorder who had psychotherapy of various sorts for an average of 11 months, after which time 48.8% did not respond to treatment [79]. An older (1995) report indicated that psychotherapy may be suboptimal for about 25%, although that was not a formal study [80]. To the query, ‘at any time during the past three years did you experience stress or other emotional problems for which you sought help…’, 2900 persons responded that they had seen a mental health professional but after 7–11 months of treatment, 25% of them perceived an inadequate, overall improvement. Combination treatment with psychotherapy plus pharmacotherapy may provide more beneficial results than treatment with either modality alone, shown by studies of patients with Obsessive Compulsive Disorder, as reviewed by Romanelli et al. [81]. Four studies of those involved 118 subjects receiving combination treatment and 125 subjects receiving pharmacotherapy alone; they showed an average standardized mean difference (SMD) of 0.76 (SMD of 0.80 is regarded as showing good efficacy). Another five of those studies that involved 92 subjects receiving combination treatment versus 93 receiving psychotherapy alone, showed an average SMD of 0.47 (SMD of 0.50 is regarded as showing moderate efficacy).

Mental symptoms, primarily disturbed cognition, are among the most disabling features of Long Covid. As presented in the text of this article, those symptoms result from impaired function of astrocytes, oligodendrocytes, neurons, and endothelial cells plus increased activity of microglia. Two drugs, lithium and fluoxetine, each benefit all of those cell-types; for depth of coverage and reduction of dosages, they should be administered together. Lithium should be used in a reduced dosage to target a serum level of 0.25–0.50 mmol/l, which was shown to provide some benefit in Alzheimer’s dementia [82]. Fluoxetine should be administered in the low dosage of 10 mg daily.

Evidence shows that low dosages of lithium and fluoxetine, administered in combination, should be well-tolerated and cause few, if any, serious side effects. Bauer et al., identified 110 patients who were receiving fluoxetine together with lithium [83]. These patients were compared with a group of patients who were not on lithium therapy. ≥ 90% in both groups used fluoxetine 20 mgs daily. Adverse events (AE) in the two groups were non-significantly different: in 21.8% during fluoxetine treatment and in 30.9% during treatment with the combination of fluoxetine/lithium (P = 0.13), and there were no serious adverse events (SAE); nor were there any statistically significant differences in any of the AE. Far higher doses than those suggested here, were administered by Fava and Alpert, who reported 34 patients who used fluoxetine 20 mg plus lithium 300–600 mg/day [84]. There were no SAE but many AE, including gastrointestinal distress in 50.0%, dry mouth in 38.2%, insomnia in 35.3%, sedation or fatigue in 32.4%, and headache in 26.5%. In brief, high dose fluoxetine with high dose lithium would cause a high drop-out rate due to AE; but as suggested here, low dosage of these two drugs, administered in combination, should cause no AE and have a low drop-out rate.

Some limitations and concerns must be considered before it may be held that administration of lithium with fluoxetine might be effective treatment for Long Covid. (1) Since the thesis presented here, relies on changed reactions in brain cell-types and their relation to cognition and emotional impairments in Long Covid, it is important to note that cognition and emotion are effectively integrated across several brain regions and it is not certain that the changed brain cell-types are present in all of them or, if present in one region but not another, how they travel the distance to an area where they are not so-changed. Pessoa has reviewed these regions, that include the hypothalamus, basal forebrain, amygdala, and areas of the neocortex (including prefrontal cortex, cingulate cortex, orbito-frontal cortex, and anterior insula), all of which have multiple projections and mutual interactions so may have long distances of separation [85]. (2) Much of the information cited in this article to support the presented hypothesis, was obtained from studies in rodents, whose brains differ from those of humans in multiple ways. As compared with rodents, human brains have more cortical fields which have more neurons, and specific cortical fields are associated with unique behavioral specializations such as language. Humans have unique types of neurons, and more varied interneurons, and human brain development involves several features of the genome that are unique to humans [86]. (3) This article focuses on brain cell-types but it must be acknowledged that there are many other risk factors for Long Covid. E.g., in 456 422 participants from the UK Biobank, Baranova et al. performed a Mendelian randomization analysis to examine relationships between COVID-19 hospitalization and health conditions, finding that a set of body fat-related traits, maternal smoking around birth, basal metabolic rate, lymphocyte count, peripheral enthesopathies and allied syndromes, blood clots in the legs, and arthropathy, are causal risk factors for severe COVID-19, while higher education attainment, physical activity, asthma, and never smoking status protect against the illness [87]. In order for these to affect mental functions, they must relate to brain cell-types, although the molecular connections are unclear. (4) It is interesting that in other analyses, severe COVID-19 was associated with an 11% increased risk for schizophrenia, suggesting that schizophrenia should be assessed as one of the post-COVID-19 sequelae [88]; and a small increase in risk for AD [89]. That raises the possibility that the changes in brain cell-types associated with severe Covid might also be related to the pathogenesis of schizophrenia and Alzheimer’s dementia. (5) It must also be acknowledged that the hypothesis proposed in this article is a theoretical construct and, as indicated below, its validity must be tested in a clinical trial.

## Clinical trial is required to validate benefit from the suggested drugs

The benefit of low dosages of fluoxetine and lithium for the mental symptoms of Long Covid, should be tested in a randomized clinical trial. Such a trial could have four arms: (1) fluoxetine 10 mg/day; (2) lithium 75–150 mg/day targeting a blood level of 0.25–0.5 mmol/l; (3) a combination of fluoxetine and lithium in the above dosages; (4) placebo. Participants must have had documented infection by SARS-2. Exclusionary criteria would be: participants must not take any other psychoactive drugs; uncontrolled diabetes; uncontrolled hypertension (BP ≥ 140/85); chronic kidney disease with eGFR < 60); untreated hepatitis C. Duration of treatment would be 12 weeks. Symptoms should be followed by using, first at baseline then monthly, appropriate tests of cognition.

## Conclusions and summary

1. Mental impairment is a serious symptom of Long Covid.
2. The brain cell-types that underpin those mental symptoms include astrocytes, oligodendrocytes, endothelial cells and microglia.
3. Both lithium and fluoxetine address all of the affected cell-types.
4. In those patients with suboptimal response to psychotherapy, a clinical trial using low dosages of both lithium and fluoxetine should test the validity of using the two drugs in combination for treatment of the mental impairment of Long Covid.
